# Apoptosis, senescence and cancer (cancer drug discovery and development)

**DOI:** 10.1038/sj.bjc.6604944

**Published:** 2009-03-03

**Authors:** A Young

**Affiliations:** 1 Cellular Senescence and Tumour Suppressors Laboratory, Cancer Research UK, Cambridge Research Institute, Robinson Way, Cambridge CB2 0RE, UK

As part of the Cancer Drug Discovery and Development series, this book aims to explain basic biological processes and signalling pathways and to link that knowledge to the anticancer drugs associated with those pathways. Each chapter begins with a detailed introduction to the basic science of the specific process, pathway or drug family before entering into their clinical applications. The book is divided into six parts and, although not officially stated, these roughly divide into two halves. The first half deals with the mechanisms of apoptosis and other modes of cell death; telomeres and senescence; and the DNA damage response. In addition to apoptosis, other modes of cell death dealt with within this half of the book include alternative cancer therapy targets such as autophagy, anoikis and mitotic catastrophe. The clinical aspects of the earlier chapters nicely links to the second half of the book, which is more pharmacologically/therapeutically orientated with three parts dealing with tumour resistance and sensitisation, established cancer therapies and developing cancer therapies. A flavour of the chapters in this half of the book include, ‘perturbation of cellular functions by Topoisomerase II inhibitors’ and ‘monoclonal antibodies in lymphomas’. With some 28 chapters, the book covers a wide range of topics within the title's remit, from information on the mechanisms of apoptosis to antimetabolite and tyrosine kinase inhibitor therapies.

The book is generally well written and extremely informative. The information within is as up to date as can be expected from this kind of book, but the reader should bear in mind the speed at which these fields are rapidly developing. The book engagingly details the history of each area and the main studies involved in forming that history. This gives the reader a broad understanding of the background to the particular area of research or therapy being discussed. On the whole, chapters are well referenced and provide easy access to the relevant literature for further reading. My only disappointment in the book was the under-representation of senescence: only a couple of the 28 chapters are wholly concerned with senescence and there are limited references to senescence in additional chapters.

I have certainly learnt a lot from reading this book. Having been previously solely involved in basic research, I have now moved into a translation research cancer centre. I therefore found the information on the therapeutic applications of what I had previously studied in terms of cell/organism function very useful. At £104 perhaps this book might be a little too expensive for an individual's collection, but I would have no hesitation in recommending it to a laboratory/clinical group or library for purchase.

